# Prevalence of delayed diagnosis of acute ischemic stroke in an acute care hospital: A single‐center cross‐sectional study in Japan

**DOI:** 10.1002/jgf2.440

**Published:** 2021-04-05

**Authors:** Chika Takarada, Junpei Komagamine, Tsutomu Mito

**Affiliations:** ^1^ Department of Internal Medicine National Hospital Organization Tochigi Medical Center Utsunomiya Japan

**Keywords:** delayed diagnosis, ischemic stroke

## Abstract

**Backgrounds:**

Given the short therapeutic window for evidence‐based therapies such as thrombolysis and endovascular treatment, it is important to immediately diagnose ischemic stroke. We investigated the prevalence of missed ischemic stroke diagnoses at initial contact and the proportion of potentially treatable patients without a delayed diagnosis.

**Methods:**

A cross‐sectional study was conducted. A total of 408 consecutive patients hospitalized due to acute ischemic stroke were included. The primary outcome was a delayed diagnosis of ischemic stroke at initial contact. A diagnosis of stroke was judged to be delayed unless physicians made a diagnosis and initiated treatment for ischemic stroke during the initial contact. The secondary outcome was ischemic stroke with a missed therapeutic window for effective treatment due to delayed diagnosis.

**Results:**

The median patient age was 78 years old, and the median time from onset to presentation was nine hours. A diagnosis of stroke was deemed delayed in 49 (12.0%) patients. In the multivariable analysis, presentation 48 hours or more after stroke onset (OR 2.45) and the improvement of neurological symptoms prior to presentation (OR 3.11) were independently associated with delayed diagnosis of ischemic stroke. Opportunities for effective treatment were missed in 18 (36.7%) of the 49 delayed diagnosis cases, although no patients missed opportunities for thrombectomy due to delayed diagnosis.

**Conclusions:**

Even in the modern era, one out of every eight ischemic stroke cases was missed at the initial visit, and one‐third of missed stroke cases might be candidates for effective treatment without diagnostic delay.

## Graphical abstract

Even in the modern era, one out of every eight ischaemic stroke cases was missed at the initial visit, and one‐third of missed stroke cases might be candidates for effective treatment without diagnostic delay.

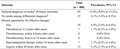

## 1 INTRODUCTION

Evidence‐based therapies have been developed to improve the prognosis of acute ischemic stroke in the last 30 years.^1^, ^2^, ^3^, ^4^, ^5^, ^6^, ^7^, ^8^, ^9^, ^10^, ^11^, ^12^, ^13^, ^14^, ^15^ To provide these effective therapies, the time from stroke onset to diagnosis is critical.^6^, ^16^ Current guidelines recommend intravenous thrombolysis for acute ischemic stroke within 4.5 hours of symptom onset and endovascular thrombectomy for acute ischemic stroke by large‐vessel occlusion within 6‐24 hours of symptom onset.^17^ Therefore, accurate diagnosis for stroke within the therapeutic window for these interventions is important.

A recent meta‐analysis regarding the prevalence of misdiagnosed ischemic stroke reported that 10%‐20% of acute ischemic stroke cases were initially misdiagnosed at the emergency department.^18^, ^19^, ^20^, ^21^, ^22^, ^23^, ^24^, ^25^, ^26^ However, only two of all studies included in the meta‐analysis^19^ investigated a missed opportunity to administer intravenous thrombolysis within its therapeutic window due to diagnostic delay.^18^, ^26^ Moreover, few studies evaluated a missed opportunity to treat patients with endovascular thrombectomy within its therapeutic window due to diagnostic delay.^18^ Therefore, further studies are needed to determine the frequency of acute ischemic stroke cases that miss an opportunity to receive these interventions due to diagnostic delay in real‐world practice. In addition, the quality of the diagnostic reference standard for ischemic stroke was not high enough in most past studies.^19^ Therefore, more studies investigating the prevalence of misdiagnosis of ischemic stroke by using diagnostic reference standards with a higher quality are also needed.

In Japan, no study has ever been conducted to investigate the prevalence and characteristics of delayed diagnosis of ischemic stroke, although one Japanese retrospective study examined the misdiagnosis of cerebellar infarction at admission.^27^ Moreover, given the limited access to board‐certified neurologists in Japan,^28^ knowing the prevalence of misdiagnosed stroke in Japanese hospitals without neurologists is important. Thus, we conducted a retrospective cross‐sectional study to investigate the prevalence and risk factors of misdiagnosed ischemic stroke in an acute care hospital in Japan.

## 2 METHODS

### 2.1 Study setting and design

A retrospective observational study (from April 2017 to July 2019) and prospective observational study (from August 2019 to October 2020) were conducted by reviewing medical electronic records to determine the proportion of delayed diagnosed stroke among acute ischemic stroke patients hospitalized in our hospital. Our hospital has no ward neurologists. Although consultation with a board‐certified neurologist from another hospital once per week is possible, consultation with a neurologist at an emergency care setting is impossible. In our hospital, patients with suspected acute stroke are initially cared for by either internists or neurosurgeons in outpatient settings, including in the emergency department, although patients who are suspected of having a stroke and are transferred by emergency medicine service personnel are directly treated by neurosurgeons. Then, patients diagnosed with acute ischemic stroke in the outpatient setting are hospitalized in either the internal medicine or the neurosurgery ward of our hospital. Stroke patients requiring surgery or interventional radiology are hospitalized in a neurosurgery ward and are cared for by neurosurgeons. Similar to our hospital, stroke care by internists is common in Japan because board‐certified neurologists are unavailable for stroke care in approximately half of Japanese hospitals.^28^, ^29^ The protocol of this study was approved by the institutional Medical Ethics Committee. This research was conducted in accordance with the Ethical Guidelines for Epidemiological Research in Japan and was carried out in accordance with the principles of the Declaration of Helsinki. The need to obtain individual informed consent was formally waived by the institutional Medical Ethics Committee because de‐identified data were collected without contacting the patients.

### 2.2 Inclusion and exclusion criteria

All patients included in the admission patient list for internal medicine, neurosurgery, and otolaryngology in our hospital were screened by reviewing their charts. Subjects were included in the study if they were hospitalized due to acute ischemic stroke and had an ischemic stroke confirmed by magnetic resonance imaging (MRI). The imaging results were confirmed by radiologists. If it was difficult to judge whether a correlation between clinical findings and MRI findings was relevant, a board‐certified neurologist judged whether the diagnosis of the case was truly acute ischemic stroke by reviewing the patient's chart. Patients who were suspected to have or diagnosed with ischemic stroke at other hospitals were excluded. Transient ischemic attack and diffusion‐weighted imaging‐negative acute ischemic stroke were also excluded.

### 2.3 Data collection and patient characteristics

Physicians reviewed the electronic medical records and retrieved information on patient age, gender, residence before index admission, past medical history, medication use, time to an initial hospital visit from symptom onset, symptoms, neurological findings, and prognosis. For information on the symptoms and neurological findings, only information documented at the initial visit and at an outpatient setting or emergency room was reported. In addition, with regard to relevant past medical history, symptoms, and neurological findings, if data were missing, they were recorded as absent in the patient. Therefore, there were no missing data for these variables. The National Institutes of Health Stroke Scale (NIHSS) scores at presentation and modified Rankin Scale (mRS) scores before stroke and at discharge were also outlined. If they were not recorded in the medical electronic records, these scores were calculated by using chart review. A past study reported that scoring for NIHSS based on retrospective chart review was reliable.^30^

### 2.4 Outcome measures

The primary outcome was delayed diagnosis of ischemic stroke at the initial visit to our hospital. A diagnosis of ischemic stroke was judged to be “delayed” unless physicians made a diagnosis of ischemic stroke during the initial contact. For cases in which physicians did not diagnose stroke but documented stroke in the differential diagnosis, a diagnosis of ischemic stroke was judged to be delayed unless treatments for acute ischemic stroke were initiated during the initial contact. Treatments included antiplatelet therapy, thrombolysis, and thrombectomy. The need for treatment for ischemic stroke and documentation of stroke as a differential diagnosis was added because the cases in which physicians did not strongly suspect stroke were included as cases of delayed diagnosis. The primary outcomes were verified by at least two investigators.

The secondary outcome was ischemic stroke with a missed therapeutic window for effective interventions due to delayed diagnosis. Based on the guidelines or past randomized controlled trials, effective interventions for stroke were defined as any of the following: (a) intravenous alteplase within 4.5 hours of symptom onset of ischemic stroke^4^, ^5^; (b) mechanical thrombectomy for anterior circulation ischemic stroke within 6 hours of symptom onset^17^; (c) mechanical thrombectomy for anterior circulation ischemic stroke within 6‐24 hours of symptom onset^15^; (d) dual antiplatelet therapy (DAPT) for noncardiogenic ischemic stroke within 24 hours of symptom onset^31^, ^32^, ^33^; and (e) aspirin for ischemic stroke within 48 hours of symptom onset.^1^, ^2^, ^3^ Based on the time of presentation at our hospital and the time of definite diagnosis for ischemic stroke, a stroke was judged to miss the therapeutic window for effective treatments due to delayed diagnosis if a diagnosis for ischemic stroke could be made within the therapeutic windows for these six interventions without diagnostic delay. However, patients with ischemic stroke that was missed at initial contact but subsequently diagnosed within their therapeutic window after hospital admission were judged to receive effective interventions within the therapeutic windows. More detailed information on the criteria for a possible candidate for effective interventions for stroke is shown in the supplementary file (Text S1). Another secondary outcome was misdiagnosed stroke defined based on the same criteria used in the previous study.^18^ A stroke was judged to be missed if physicians did not initially consider stroke in the differential diagnosis during first contact, or the diagnosis was delayed, causing the patient to miss the therapeutic window for thrombolytic therapy. Two investigators independently assessed these secondary outcomes by reviewing the charts based on these criteria, and discrepancies were resolved by discussions between the two investigators. The rate of outcome assessment agreement between the two investigators before discussion was 75.5% (kappa 0.53).

### 2.5 Statistical analysis

Assuming that the proportion of delayed diagnosis of ischemic stroke was 20% based on past studies,^18^, ^19^ approximately 400 ischemic stroke patients were needed to provide a precision of 3% for the calculation of the 95% confidence interval (CI) of the primary outcome.

We used descriptive statistics to report the baseline characteristics of the study population. The 95% CIs were calculated for the primary and secondary outcomes. The comparison for all variables between patients with and without delayed diagnosis of ischemic stroke was conducted by using Fisher's exact test for categorical variables and the Mann‐Whitney *U* test for continuous variables. All tests were two‐sided, and the level of statistical significance was set at 5%. Based on a previous study,^18^ logistic regression models were computed by including all variables with a significant *P*‐value from univariable analysis, excluding variables that were identified in <5% of the total population to avoid unstable models. At this stage, we included the twelve variables: prestroke modified Rankin Scale, NIHSS at presentation, time to presentation from onset, posterior circulation, unilateral weakness, dysarthria, facial palsy, sensory sign, dizziness, nausea, improvement of neurological signs, and care by neurosurgeons. Then, variables were removed one‐by‐one with a backward stepwise method until all remaining variables had a *P*‐value < .4. After excluding two variables (posterior circulation and dizziness), the final model included ten variables. Stata version 15 (LightStone) was used for these analyses.

## 3 RESULTS

During the study period, 706 patients were hospitalized due to acute ischemic stroke. After excluding 298 patients, a total of 408 patients were included in the present study (detailed information is shown in the supplementary file; Figure S1). In all included patients, the median age was 78 years (interquartile range (IQR) 70‐85 years old), 170 (41.7%) were women, 100 (24.5%) had a history of stroke, 43 (10.5%) had dementia, and the median prestroke mRS score was 0 (IQR 0‐1) (Table 1 and Table S1). The most common symptoms or signs were unilateral weakness (n = 276, 67.7%), followed by dysarthria (n = 199, 48.8%) and facial palsy (n = 145, 35.5%). The median time to presentation from the time that the patient was last known to be well was 9 hours (IQR 2‐24), and the median NIHSS score at presentation was 4 (IQR 2‐10).

**TABLE 1:** Baseline characteristics of the 408 patients with acute ischemic stroke^a^

Characteristics	Total	Delayed diagnosis		*P*‐value^b^
		Yes (n = 49)	No (n = 359)	
Age, median (IQR)	78 (70‐85)	81 (73‐87)	77 (70‐84)	.13
Female gender	170 (41.7)	20 (40.8)	150 (41.8)	1
Nursing home resident	23 (5.6)	5 (10.2)	18 (5.0)	.18
Prestroke mRS scores				
Median, IQR	0 (0‐1)	0 (0‐3)	0 (0‐1)	.03
Less than three points	344 (84.3)	35 (71.4)	309 (86.1)	.01
Ambulance use	317 (77.7)	37 (75.5)	280 (78.0)	.72
Interval between time that patient was last known to be well and presentation				
Median hours (IQR)	9 (2‐24)	17 (2‐54)	9 (2‐23)	<.001
More than 48 h	63 (15.4)	17 (34.7)	46 (12.8)	<.001
Past medical history				
Hypertension	276 (67.7)	34 (69.4)	242 (67.4)	.87
Diabetes mellitus	102 (25.0)	12 (24.5)	90 (25.1)	1
Dyslipidemia	104 (25.5)	16 (32.7)	88 (24.5)	.22
Atrial fibrillation	55 (13.5)	11 (22.5)	44 (12.3)	.07
Ischemic heart disease	22 (5.4)	4 (8.2)	18 (5.0)	.32
Stroke	100 (24.5)	16 (32.7)	84 (23.4)	.16
Dementia	43 (10.5)	7 (14.3)	36 (10.0)	.33
Symptom and neurological findings				
Headache	19 (4.7)	6 (12.2)	13 (3.6)	.02
Nausea or vomiting	40 (9.8)	14 (28.6)	26 (7.2)	<.001
Vertigo, dizziness, or imbalance	34 (8.3)	11 (22.5)	23 (6.4)	<.001
Auditory symptom	5 (1.2)	1 (2.0)	4 (1.1)	.47
Syncope or transient LOC	10 (2.5)	6 (12.2)	4 (1.1)	<.001
Seizure	7 (1.7)	4 (8.2)	3 (0.8)	.005
Unilateral weakness	276 (67.7)	8 (16.3)	268 (74.7)	<.001
Bilateral weakness	37 (9.1)	8 (16.3)	29 (8.1)	.07
Dysarthria	199 (48.8)	8 (16.3)	191 (53.2)	<.001
Facial palsy	145 (35.5)	2 (4.1)	143 (39.8)	<.001
Sensory	91 (22.3)	2 (4.1)	89 (24.8)	<.001
Neglect	53 (13.0)	2 (4.1)	51 (14.2)	.07
Aphasia	109 (26.7)	8 (16.3)	101 (28.1)	.09
Dysmetria	15 (3.7)	8 (16.3)	12 (3.3)	.41
Ataxia	50 (12.3)	7 (14.3)	43 (12.0)	.64
Gaze preference	46 (11.3)	6 (12.2)	40 (11.1)	.81
Altered mental status	90 (22.1)	15 (30.6)	75 (20.9)	.14
Disorientation	110 (27.0)	13 (26.5)	97 (27.0)	1
Vision change	29 (7.1)	1 (2.0)	28 (7.8)	.23
Tendency of neurological signs to improve until presentation	47 (11.5)	12 (24.5)	35 (9.8)	.01
NIHSS score at presentation, median (IQR)	4 (2‐10)	1 (0‐6)	4 (2‐11)	.01
Physicians caring for the patients				
Resident	139 (34.1)	19 (38.8)	120 (33.4)	.52
Internists	168 (41.2)	31 (63.3)	137 (38.2)	.001
Neurosurgeons	210 (51.5)	12 (24.5)	198 (55.2)	<.001
Brain imaging performed at initial contact				
Computed tomography	243 (59.6)	35 (71.4)	208 (57.9)	.09
Magnetic resonance imaging	323 (79.2)	9 (18.4)	314 (87.5)	<0.001
Median time to stroke diagnosis from presentation, days	NA	1 (1‐3)	NA	NA
Location of ischemic stroke				
Anterior circulation	274 (67.2)	24 (49.0)	250 (69.6)	.01
Posterior circulation	104 (25.5)	24 (49.0)	80 (22.3)	<.001
Both	30 (7.4)	1 (2.0)	29 (8.1)	.24
Thrombolysis	53 (13.0)	0 (0.0)	53 (14.8)	.001
Thrombectomy	34 (8.3)	0 (0.0)	34 (9.5)	.02
Median days of hospital stay (IQR)	24 (13‐39)	25 (10‐39)	24 (14‐39)	.54
In‐hospital mortality	29 (7.1)	4 (8.2)	25 (7.0)	.77
Poststroke mRS scores at discharge				
Median (IQR)	4 (2‐4)	4 (1‐4)	4 (2‐4)	.69
Less than three points	139 (34.1)	17 (34.7)	122 (34.0)	1
Destination after discharge				
Home	165 (40.4)	22 (44.9)	143 (39.8)	.54
Nursing home	31 (7.6)	5 (10.2)	26 (7.2)	.4
Rehabilitation facilities	144 (35.3)	12 (24.5)	132 (36.8)	.11

Abbreviations: IQR, interquartile range; LOC, loss of consciousness; mRS, modified Rankin Scale; NA, not applicable; NIHSS, National Institutes of Health Stroke Scale.

^a^ Values are expressed as the number with the percentage of the total number, unless otherwise stated.

^b^ Comparisons between patients with and without delayed diagnosis of ischemic stroke were performed by using Fisher's exact test for categorical variables and the Mann‐Whitney *U* test for continuous variables. The level of statistical significance was set at 5%.

For the primary outcome, a diagnosis of 49 ischemic strokes (12.0%; 95% CI 8.8%‐15.2%) among all cases was judged to be delayed (Table 2). Of those, the median time from first contact to stroke diagnosis was 1 day (IQR 1‐3). For the secondary outcome, the proportion of patients for whom stroke was not considered among the differential diagnoses was 6.1% (95%CI 3.8%‐8.5%). Of the 49 ischemic stroke cases with a delayed diagnosis, 18 (36.7%) were judged to have missed opportunity for effective intervention. Seven cases, seven cases, and 13 cases were judged to have missed the opportunities for thrombolysis 4.5 hours after onset, DAPT within 24 hours after onset, and aspirin within 48 hours after onset, respectively. However, no stroke case had missed opportunities for thrombectomy within 6 hours after onset and thrombectomy from 6 to 24 hours after onset. The most common initial diagnosis at first contact in the ischemic stroke patients with delayed diagnoses was epilepsy (n = 8, 16.3%), followed by dizziness (n = 6, 12.2%) and head trauma or concussion (n = 4, 8.2%) (Table 3).

**TABLE 2:** Prevalence of delayed diagnosis of ischemic stroke among the 408 ischemic stroke patients

Outcome	Total	Prevalence, 95% CI
	(n = 408)	
Delayed diagnosis of stroke^a^ (primary outcome)	49	12.0% (8.8%‐15.2%)
No stroke among differential diagnosis^b^	25	6.1% (3.8%‐8.5%)
Missed opportunity for effective therapy^c^		
Any	18	4.4% (2.4%‐6.4%)
Thrombolysis 4.5 h after onset	7	1.7% (0.5%‐3.0%)
Thrombectomy within 6 h after onset	0	0.0% (NA)
Thrombectomy from 6 to 24 h after onset	0	0.0% (NA)
Dual antiplatelet therapy within 24 h after onset	7	1.7% (0.5%‐3.0%)
Aspirin within 48 h after onset	13	3.2% (1.5%‐4.9%)

Abbreviations: CI, confidence interval; NA, not applicable.

^a^ A diagnosis of ischemic stroke was judged to be “delayed” unless physicians made a diagnosis and initiated treatment for ischemic stroke during the initial contact.

^b^ A stroke was judged to be missed if physicians did not initially consider stroke in the differential diagnosis during first contact or if the diagnosis was delayed causing the patient to miss the therapeutic window for thrombolytic therapy.

^c^ A stroke was judged to miss the therapeutic window for effective treatments due to delayed diagnosis if a diagnosis for ischemic stroke could be made within the therapeutic windows for these six interventions without diagnostic delay.

**TABLE 3:** Initial diagnosis of the 49 patients with a delayed diagnosis of ischemic stroke

Diagnosis	Number (n = 49)
Epilepsy	8
Vertigo or dizziness	6
Head trauma or concussion	4
Benign paroxysmal positional vertigo	3
Altered mental status	3
Dementia or delirium	3
Suspected transient ischemic stroke	2
Ataxia	2
Heat stroke	2
Drug adverse event	2
Dehydration	2
Transient weakness	2
Gastroenteritis	1
Vomiting	1
Hypertension	1
Anorexia	1
Heart failure	1
Vestibular neuritis	1
Suspected stroke	1
Pneumonitis	1
Hemiparesis	1
Alcohol intoxication	1

A comparison of clinical features was performed between patients with and without a delayed diagnosis of ischemic stroke, and a prestroke mRS score greater than two points and more than 48 hours from the time that the patient was last known to be well to presentation were significantly associated with a delayed diagnosis of ischemic stroke. Symptoms or signs associated with a delayed diagnosis of ischemic stroke were headache, nausea or vomiting, dizziness, transient loss of consciousness, and seizure, while those associated with an accurate diagnosis of ischemic stroke were unilateral weakness, dysarthria, facial palsy, and sensory sign. With regard to the other factors, lower NIHSS scores at presentation, improvement of neurological signs until presentation, and posterior circulation stroke were associated with a delayed diagnosis of ischemic stroke, while care by neurosurgeons and anterior circulation stroke were associated with an accurate diagnosis. There was no significant difference in in‐hospital mortality between patients with and without a delayed diagnosis of ischemic stroke.

A multivariable analysis revealed that presentation 48 hours or more after stroke onset (OR 2.45, 95% CI 1.02‐5.94) and improvement of neurological symptoms prior to presentation (OR 3.11, 95% CI 1.24‐7.76) were independently associated with an increased risk of delayed diagnosis of ischemic stroke (Table 4). Conversely, a prestroke mRS score less than three points (OR 0.30, 95% CI 0.11‐0.80) and unilateral weakness (OR 0.15, 95% CI 0.06‐0.36) were independently associated with a decreased risk of delayed diagnosis of ischemic stroke.

**TABLE 4:** Summary of the multivariable logistic regression results^a^ to predict the delayed diagnosis of ischemic stroke

Variables	Odds ratio (95% CI)	*P*‐value
Care by neurosurgeons	0.47 (0.21‐1.06)	.07
Prestroke modified Rankin Scale < 3	0.30 (0.11‐0.80)	.02
NIHSS at presentation	0.97 (0.91‐1.03)	.29
Presentation at more or 48 h from the stroke onset	2.45 (1.02‐5.94)	.046
Unilateral weakness	0.15 (0.06‐0.36)	<.001
Dysarthria	0.48 (0.18‐1.24)	.13
Facial palsy	0.25 (0.05‐1.17)	.08
Sensory sign	0.30 (0.06‐1.47)	.14
Nausea or vomiting	2.54 (0.95‐6.77)	.06
Improvement of neurological signs prior to presentation	3.11 (1.24‐7.76)	.02

Abbreviation: NIHSS, National Institutes of Health Stroke Scale.

^a^ Variables were removed one‐by‐one until all remaining variables had a *P*‐value of <.4 by using a backward stepwise method. The following variables were used: prestroke modified Rankin Scale, NIHSS at presentation, time to presentation from onset, posterior circulation, unilateral weakness, dysarthria, facial palsy, sensory sign, dizziness, nausea, improvement of neurological signs, and care by neurosurgeons. The level of statistical significance was set at 5%.

## 4 DISCUSSION

Our finding is consistent with that of past similar studies reporting that 10%‐20% of ischemic stroke cases were missed in the emergency department.^18^, ^19^, ^21^, ^23^, ^24^, ^26^ This finding implies that the prevalence of delayed diagnosis of ischemic stroke has not improved during the past decade. However, the definition of our primary outcome was somewhat different from that of past studies.^18^, ^21^, ^23^, ^24^, ^26^ In past studies,^18^, ^21^, ^23^, ^24^, ^26^ a stroke was not judged to be missed if only a stroke was suspected or considered as a differential diagnosis. If we compare our results with those of a previous study^18^ by using the same criteria for a definition of missed ischemic stroke, the proportion of missed ischemic stroke in the present study was 6.1%, which is much lower than that of past studies.^18^, ^23^, ^24^, ^26^ Moreover, most past similar studies were conducted in hospitals with ward neurologists.^18^, ^21^, ^23^, ^24^ Therefore, our finding in the hospital without ward neurologists is somewhat encouraging. Nonetheless, given that a single‐center study design limits the generalizability of our findings, further study is needed to investigate the trends in and prevalence of delayed diagnosis of ischemic stroke in other Japanese hospitals and other countries using the same inclusion criteria and outcome measures.^19^

In the present study, a substantial proportion of ischemic stroke patients missed opportunities for effective therapy within a therapeutic window due to diagnostic delay. However, ischemic stroke patients who missed opportunities for thrombolysis due to diagnostic delay were uncommon, and there were no patients who missed opportunities for thrombectomy due to diagnostic delay. Our findings are consistent with the findings of a past study showing that the proportion of patients who missed opportunities for thrombolysis due to diagnostic delay in hospitalized patients with ischemic stroke was 1.1%.^26^ For missed opportunities for thrombectomy due to diagnostic delay, Arch et al^18^ reported that the proportion of ischemic stroke patients who arrived at symptom onset within the therapeutic window of thrombectomy but had a missed opportunity was 2.4%. However, given that indications of thrombectomy for ischemic stroke other than a time window from symptom onset were not considered in the past study, it is possible that the past study^18^ overestimated the missed opportunities for thrombectomy due to diagnostic delay. Therefore, the results of our study and the past study^18^ implicate that there are few patients with ischemic stroke who meet the criteria at initial contact and miss opportunities for thrombectomy due to diagnostic delay in real‐world practice. Given that a therapeutic target lesion of thrombectomy is an occlusion of the proximal middle cerebral artery and internal carotid artery and anterior circulation stroke has a lower risk of delayed diagnosis than posterior circulation stroke,^18^, ^19^, ^26^ ischemic stroke patients who meet the indicated criteria for thrombectomy may be less likely to be misdiagnosed. For missed opportunities for DAPT and aspirin due to diagnostic delay in hospitalized patients with ischemic stroke, our results were not compared with those of past studies due to the absence of past studies investigating these outcomes. Given the efficacy of DAPT for minor ischemic stroke within 24 hours after onset,^31^, ^32^, ^33^ it seems problematic that a substantial proportion of ischemic stroke patients had missed opportunities for DAPT due to diagnostic delay. Because only a few studies have been conducted to investigate the proportion of ischemic stroke patients who had missed opportunities for these interventions due to misdiagnosis, further studies are warranted.

Multivariable analysis revealed that presentation 48 hours or more after stroke onset, prestroke mRS scores greater than two points, and improvement of neurological signs prior to presentation were independent risk factors for the misdiagnosis of ischemic stroke, while unilateral weakness significantly reduced the risk of delayed diagnosis of ischemic stroke. These results are consistent with those of past studies regarding prestroke mRS scores greater than two points^20^ and unilateral weakness.^18^, ^26^ Our study was the first to investigate the effect of improvement of neurological signs from onset on delayed diagnosis of ischemic stroke. Symptom improvement prior to presentation was an independent risk factor for the misdiagnosis of ischemic stroke. Improvement of neurological signs prior to presentation might make accurate diagnosis difficult or mislead physicians to misdiagnose the stroke as a less dangerous illness.

Our findings suggest that the misdiagnosis of ischemic stroke is still common even in the modern era. Although missed opportunities for thrombolysis and thrombectomy due to delayed diagnosis are infrequent, a substantial proportion of ischemic stroke patients may miss opportunities for DAPT. To avoid missing the therapeutic window for effective interventions for ischemic stroke due to delayed diagnosis, patients with disabilities or whose neurological signs improve prior to presentation should be carefully treated. Moreover, given that vestibular disease and epilepsy were the most common initial diagnoses among stroke patients with delayed diagnosis, some strategies to identify ischemic stroke accurately among patients presenting with dizziness, nausea, or seizure are needed. For example, an approach based on timing and triggers might be effective for patients presenting with dizziness.^34^ In addition, an ideal strategy is to ensure the availability of consulting neurologists 24 hours a day in the emergency department.

Our study has several strengths. This study was the first in Japan to determine the prevalence of delayed diagnosis of acute ischemic stroke. Furthermore, this was also the first study to investigate the prevalence of misdiagnosed ischemic stroke that missed an opportunity to receive interventions by DAPT and aspirin treatments within an appropriate therapeutic window. The quality of the diagnostic reference standard used in the present study for ischemic stroke is high based on the criteria of a previous meta‐analysis,^19^ although that of most past similar studies was moderate.^18^, ^21^, ^23^, ^26^ In addition, to minimize missing eligible ischemic stroke patients whose discharge diagnoses were recorded incorrectly as other diseases, we performed a chart review of all hospitalized patients during the study period. Nonetheless, several limitations should be mentioned. First, data were collected retrospectively. Therefore, the information retrieved in this study was not accurate. For example, a past study reported that the mRS scores based on the retrospective chart review were not reliable.^35^ However, a study in which data were collected prospectively to examine the misdiagnosis of acute stroke would not reflect real‐world clinical practice because it would introduce the Hawthorne effect.^36^ Second, a single‐center study design limits the generalizability of our findings. Third, stroke patients who were confirmed by brain computed tomography or who were not diagnosed by brain MRI were excluded. Fourth, stroke patients who were missed at initial care in our hospital but diagnosed in other hospitals were not evaluated. Moreover, our study did not include stroke patients who were missed in our hospital but improved spontaneously without intervention. Therefore, the prevalence of delayed diagnosis of stroke might be underestimated in the present study. Fifth, stroke accounts for a substantial proportion of diseases that were overlooked by clinicians but were uncovered by autopsy. Therefore, some stroke patients who died in our hospital were undiagnosed during their hospital stays.^37^ Sixth, although some observational studies and subanalyses of randomized controlled trials supported that earlier intravenous thrombolysis or endovascular thrombectomy may result in a better prognosis of acute ischemic stroke,^6^, ^16^, ^38^ there have been no randomized controlled trials regarding attempts to improve the prognosis of stroke patients by lowering the proportion with a delayed diagnosis of acute ischemic stroke. Seventh, the overdiagnosis of acute ischemic stroke was not evaluated in the present study.^39^ Too much emphasis on the avoidance of a delayed stroke diagnosis may increase overdiagnosis of acute ischemic stroke, which may result in harm.^40^ Eighth, the interobserver agreement for the assessment for the secondary outcome was not adequate. Finally, the occurrence of the primary outcome was less common than we had expected. Given that the number of patients who experienced the primary outcome was approximately 50, the inclusion of ten variables in the multivariable analysis might have made the statistical model unstable.

In conclusion, one in every eight ischemic stroke cases was missed at the initial visit, and one‐third of missed strokes might be candidates for effective treatment without diagnostic delay. A prestroke mRS score greater than two points, presentation 48 hours or more after the onset of stroke, and improvement of neurological signs prior to presentation were independent risk factors for misdiagnosis of ischemic stroke. Although missed opportunities for thrombolysis and thrombectomy due to delayed diagnosis are infrequent, further efforts are required to avoid the delayed diagnosis of ischemic stroke.

## CONFLICT OF INTEREST

The authors have stated explicitly that there are no conflicts of interest in connection with this article.

## Supporting information

Supplementary Material
