# Supplementary material for: Prevalence of delayed diagnosis of acute ischemic stroke in an acute care hospital: A single‐center cross‐sectional study in Japan
Source: J Gen Fam Med. 2021 Apr 5;22(5):262–70. doi: 10.1002/jgf2.440 (PMC8411402; doi:10.1002/jgf2.440)
Supplement: Supplementary file 1 — Supplementary Material [file JGF2-22-262-s001.pdf]

Text S1. Assessment of the opportunity for effective interventions for ischaemic stroke.

Assessment of an opportunity for thrombolysis 3 hours after symptom onset.

Based on the past randomised controlled trial [1], an ischaemic stroke was judged to be a candidate for thrombolysis 3 hours after the onset unless any of the following were identified in the delayed diagnosis case: (1) haemorrhagic stroke; (2) another stroke or severe head trauma within the preceding 3 months; (3) major surgery within the preceding 14 days; (4) past history of intracranial haemorrhage; (5) rapidly improving or minor symptoms; (6) gastrointestinal haemorrhage or urinary tract haemorrhage within the preceding 21 days; (7) symptoms suggestive of subarachnoid haemorrhage; (8) seizure at the onset; (9) arterial puncture at a noncompressible site within the preceding 7 days; (10) receiving anti-coagulant therapy and elongation of partial thromboplastin time; (11) platelet count below 100000 per cubic millimetre; (12) glucose concentrations below 50 mg per decilitre or 400 mg per decilitre.

Assessment of an opportunity for thrombolysis from 3 to 4.5 hours after symptom onset.

Based on the past randomised controlled trial [2], an ischaemic stroke was judged to be a candidate for thrombolysis from 3 to 4.5 hours after the onset unless any of the following were identified in the delayed diagnosis case: (1) more than 80 years old; (2) past history of diabetes mellitus or stroke; (3) NIHSS score above 25; (4) use of anticoagulants; (5) intracranial haemorrhage; (6) seizure at the onset of stroke; (7) rapidly improving or minor symptoms; (8) major surgery or severe head trauma within the preceding 3 months; (9) platelet count below 100000 per cubic millimetre; (10) glucose concentrations below 50 mg per decilitre or above 400 mg per decilitre.

Assessment of an opportunity for thrombectomy within 6 hours after symptom onset.

Based on past randomised controlled trials and guidelines [3], an ischaemic stroke was judged to be a candidate for thrombectomy within 6 hours after the onset if all of the following were identified in the delayed diagnosis case: (1) anterior circulation stroke; (2) pre-stroke modified Rankin Scale (mRS) score below 2; and (3) NIHSS score above 5.

Assessment of an opportunity for thrombectomy from 6 to 24 hours after symptom onset.

Based on the past randomised controlled trial [4], an ischaemic stroke was judged to be a candidate for thrombectomy if all of the following were identified in the delayed diagnosis case: (1) anterior circulation stroke; (2) pre-stroke mRS below 2; (3) NIHSS score above 9; and (4) no terminal illnesses.

Assessment of an opportunity for DAPT within 24 hours after symptom onset.

Based on past randomised controlled trials [5,6], ischaemic stroke was judged to be a candidate for DAPT within 24 hours after onset if all of the following were identified in the delayed diagnosis case: (1) noncardiogenic ischaemic stroke; (2) no indications of anti-coagulant therapy; (3) NIHSS score below 4; and (4) no atrial fibrillation.

Assessment of an opportunity for aspirin within 48 hours after symptom onset.

Based on past randomised controlled trials [7,8], ischaemic stroke was judged to be a candidate for DAPT within 48 hours after onset if all of the following criteria were satisfied in the delayed diagnosis case: (1) pre-stroke mRS below 5; (2) no use of anticoagulants; and (3) no active peptic ulcer.

References

1. The National Institue of Neurological Disorders and Stroke rt-PA Stroke Study Group. Tissue plasminogen activator for acute ischemic stroke. N Engl J Med 1995;333(24):1581-7. doi:10.1056/NEJM199512143332401.
2. Hacke W, Kaste M, Bluhmki E, et al. Thrombolysis with alteplase 3 to 4.5 hours after acute ischemic stroke. N Engl J Med 2008;359(13):1317-29. doi:10.1056/NEJMoa0804656.
3. Powers WJ, Rabinstein AA, Ackerson T, et al; American Heart Association Stroke Council. 2018 Guidelines for the Early Management of Patients With Acute Ischemic Stroke: A Guideline for Healthcare Professionals From the American Heart Association/American Stroke Association. *Stroke* 2018;49(3):e46-e110. doi:10.1161/STR.0000000000000158.
4. Nogueira RG, Jadhav AP, Haussen DC, et al; DAWN Investigators. Thrombectomy 6 to 24 hours after stroke with a mismatch between deficit and infarct. *N Engl J Med* 2018;378(1):11-21. doi:10.1056/NEJMoa1706442.

5. Wang Y, Wang Y, Zhao X, et al. Clopidogrel with aspirin in acute minor stroke or transient ischemic attack. *N Engl J Med* 2013;369(1):11-9. doi;10.1056/NEJMoa1215340.
6. Johnston SC, Easton JD, Farrant M, et al; Clinical Research Collaboration, Neurological Emergencies Treatment Trials Network, and POINT Investigators. Clopidogrel and aspirin in acute ischemic stroke and high-risk TIA. *N Engl J Med* 2018;379(3):215-25. doi:10.1056/NEJMoa1800410.
7. International Stroke Trial Collaborative Group. The International Stroke Trial (IST): a randomised trial of aspirin, subcutaneous heparin, both, or neither among 19435 patients with acute ischaemic stroke. *Lancet* 1997;349(9065):1569-81. doi;10.1016/S0140-6736(97)04011-7.
8. CAST (Chinese Acute Stroke Trial) Collaborative Group. CAST: randomised placebo-controlled trial of early aspirin use in 20000 patients with acute ischaemic stroke. *Lancet* 1997;349(9066):1641-9. doi:10.1016/S0140-6736(97)04010-5.

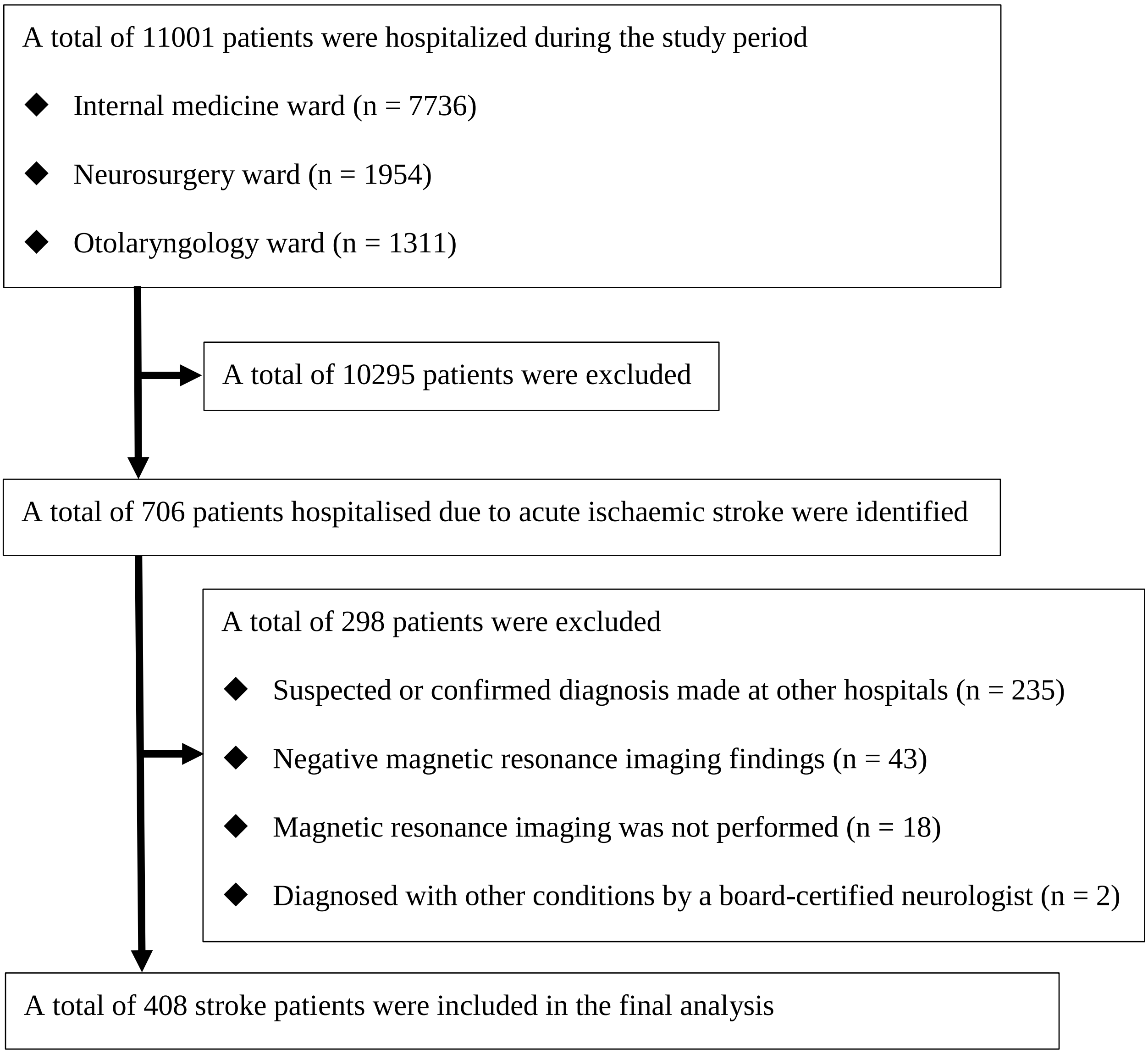

Figure S1. Flow chart of the 408 patients included in this study.

Table S1. Baseline characteristics of the 408 patients with acute ischaemic stroke[a].

**Characteristics**	**Total**	**Delayed diagnosis**		
		**Yes (n = 49)**	**No (n = 359)**	***P*-value[b]**
Age, median (IQR)	78 (70-85)	81 (73 - 87)	77 (70 - 84)	0.13
Female sex	170 (41.7)	20 (40.8)	150 (41.8)	1.00
Japanese	407 (99.8)	49 (100.0)	358 (99.7)	1.00
Current smoker	93 (22.9)	10 (20.4)	83 (23.2)	0.86
Regular drinker	93 (22.9)	6 (12.2)	87 (24.3)	0.07
Nursing home resident	23 (5.6)	5 (10.2)	18 (5.0)	0.18
Pre-stroke mRS scores				
Median, IQR	0 (0 - 1)	0 (0 - 3)	0 (0 - 1)	0.03
Less than three points	344 (84.3)	35 (71.4)	309 (86.1)	0.01
Ambulance use	317 (77.7)	37 (75.5)	280 (78.0)	0.72
Interval between time that patient was last known to be well and presentation				
Median hours (IQR)	9 (2 - 24)	17 (2 - 54)	9 (2 - 23)	< 0.001
More than 48 hours	63 (15.4)	17 (34.7)	46 (12.8)	< 0.001
Past medical history				
Hypertension	276 (67.7)	34 (69.4)	242 (67.4)	0.87
Diabetes mellitus	102 (25.0)	12 (24.5)	90 (25.1)	1.00
Dyslipidaemia	104 (25.5)	16 (32.7)	88 (24.5)	0.22
Atrial fibrillation	55 (13.5)	11 (22.5)	44 (12.3)	0.07
Ischaemic heart disease	22 (5.4)	4 (8.2)	18 (5.0)	0.32
Stroke	100 (24.5)	16 (32.7)	84 (23.4)	0.16
Dementia	43 (10.5)	7 (14.3)	36 (10.0)	0.33
Symptom and neurological findings				
Headache	19 (4.7)	6 (12.2)	13 (3.6)	0.02
Nausea or vomiting	40 (9.8)	14 (28.6)	26 (7.2)	< 0.001
Vertigo, dizziness, or imbalance	34 (8.3)	11 (22.5)	23 (6.4)	< 0.001
Auditory symptom	5 (1.2)	1 (2.0)	4 (1.1)	0.47
Syncope or transient LOC	10 (2.5)	6 (12.2)	4 (1.1)	< 0.001
Seizure	7 (1.7)	4 (8.2)	3 (0.8)	0.005
Unilateral weakness	276 (67.7)	8 (16.3)	268 (74.7)	< 0.001
Bilateral weakness	37 (9.1)	8 (16.3)	29 (8.1)	0.07
Dysarthria	199 (48.8)	8 (16.3)	191 (53.2)	< 0.001
Facial palsy	145 (35.5)	2 (4.1)	143 (39.8)	< 0.001

Sensory	91 (22.3)	2 (4.1)	89 (24.8)	< 0.001
Neglect	53 (13.0)	2 (4.1)	51 (14.2)	0.07
Aphasia	109 (26.7)	8 (16.3)	101 (28.1)	0.09
Dysmetria	15 (3.7)	8 (16.3)	12 (3.3)	0.41
Ataxia	50 (12.3)	7 (14.3)	43 (12.0)	0.64
Gaze preference	46 (11.3)	6 (12.2)	40 (11.1)	0.81
Altered mental status	90 (22.1)	15 (30.6)	75 (20.9)	0.14
Disorientation	110 (27.0)	13 (26.5)	97 (27.0)	1.00
Vision change	29 (7.1)	1 (2.0)	28 (7.8)	0.23
Tendency of neurological signs to improve until presentation	47 (11.5)	12 (24.5)	35 (9.8)	0.01
NIHSS score at presentation, median (IQR)	4 (2 - 10)	1 (0 - 6)	4 (2 - 11)	0.01
Physicians caring for the patients				
Resident	139 (34.1)	19 (38.8)	120 (33.4)	0.52
Internists	168 (41.2)	31 (63.3)	137 (38.2)	0.001
Neurosurgeons	210 (51.5)	12 (24.5)	198 (55.2)	< 0.001
Brain imaging performed at initial contact				
Computed tomography	243 (59.6)	35 (71.4)	208 (57.9)	0.09
Magnetic resonance imaging	323 (79.2)	9 (18.4)	314 (87.5)	< 0.001
Median time to stroke diagnosis from presentation, days	NA	1 (1 - 3)	NA	NA
Location of ischaemic stroke				
Anterior circulation	274 (67.2)	24 (49.0)	250 (69.6)	0.01
Posterior circulation	104 (25.5)	24 (49.0)	80 (22.3)	< 0.001
Both	30 (7.4)	1 (2.0)	29 (8.1)	0.24
Thrombolysis	53 (13.0)	0 (0.0)	53 (14.8)	0.001
Thrombectomy	34 (8.3)	0 (0.0)	34 (9.5)	0.02
Median days of hospital stay (IQR)	24 (13 - 39)	25 (10 - 39)	24 (14 - 39)	0.54
In-hospital mortality	29 (7.1)	4 (8.2)	25 (7.0)	0.77
Post-stroke mRS scores at discharge				
Median (IQR)	4 (2 - 4)	4 (1 - 4)	4 (2 - 4)	0.69
Less than three points	139 (34.1)	17 (34.7)	122 (34.0)	1.00
Destination after discharge				
Home	165 (40.4)	22 (44.9)	143 (39.8)	0.54
Nursing home	31 (7.6)	5 (10.2)	26 (7.2)	0.40
Rehabilitation facilities	144 (35.3)	12 (24.5)	132 (36.8)	0.11

[a]Values are expressed as the number with the percentage of the total number, unless otherwise stated.

[b]Comparisons between patients with and without delayed diagnosis of ischaemic stroke were performed by using Fisher's exact test for categorical variables and the Mann-Whitney U test for continuous variables. The level of statistical significance was set at 5%.

IQR, interquartile range; LOC, loss of consciousness; mRS, modified Rankin Scale; NA, not applicable; NIHSS, National Institutes of Health Stroke Scale.
